# Impact of Buzhong Yiqi Prescription on the Gut Microbiota of Patients with Obesity Manifesting Polycystic Ovarian Syndrome

**DOI:** 10.1155/2021/6671367

**Published:** 2021-03-15

**Authors:** Zhexin Ni, Wen Cheng, Jie Ding, Ruipin Yao, Danying Zhang, Dongxia Zhai, Ling Zhou, Chaoqin Yu

**Affiliations:** ^1^Department of Traditional Chinese Gynecology, Changhai Hospital, Naval Medical University, Shanghai 200433, China; ^2^Shanghai University of Traditional Chinese Medicine, Shanghai 201203, China

## Abstract

Gut microbiota disorders are closely related to polycystic ovarian syndrome (PCOS). Buzhong Yiqi prescription (BZYQ) has a significant clinical effect on the treatment of patients with obesity exhibiting PCOS and phlegm-dampness syndrome caused by spleen deficiency (SPSD). Hence, this study aimed to explore gut microbiota and fecal metabolite alterations in such patients treated with BZYQ. Fifty eligible patients with obesity manifesting PCOS and SPSD participated and agreed to undergo 3 months of BZYQ treatment. Results showed that BZYQ significantly alleviated the serum dehydroepiandrosterone sulfate (*p* < 0.001) and testosterone levels (*p* < 0.001) and markedly changed the gut microbiota structure in these patients. Furthermore, 106 differential fecal metabolites and 14 KEGG enrichment pathways were quantified. The phylum Spirochaetae and the genera *[Eubacterium]_rectale_group*, *Escherichia-Shigella*, and *Fusicatenibacter* were significantly more abundant, but *Megamonas* was significantly less abundant after treatment than before treatment. Disorders in the gut microbiota and fecal metabolites of these patients were closely related to hyperandrogenemia and insulin resistance. In conclusion, BZYQ could ameliorate the serum androgen level and had an impact on the gut microbiota and metabolites in patients with obesity manifesting PCOS and SPSD.

## 1. Introduction

Polycystic ovarian syndrome (PCOS) is a chronic disease affecting the reproductive endocrine and metabolic mechanisms. Common manifestations include different degrees of menstrual abnormalities, infertility, hair excess/loss, acne, and obesity. The incidence rate of PCOS reaches 5%–15% [1], occurring mostly in puberty and childbearing age. Unfortunately, its cause remains unclear [2]. Epidemiological data showed that approximately 53.5%–85.5% of patients with PCOS are overweight or obese, with central obesity as the most typical [3]. Obesity is the most perilous factor for insulin resistance. For patients with obesity exhibiting PCOS, weight loss remains one of the important treatments [4].

In recent years, traditional Chinese medicine (TCM) and intestinal microecology have gained considerable attention in research. Some single-herb or TCM compounds can help maintain the balance of intestinal microecology [5]. Generally, TCM is essentially used as an oral herbal decoction or other dosage forms for treating diseases. This natural therapy plays a role in the body locally or systemically through the digestive tract, the most important region for parasitizing microorganisms. The gut microbiota is closely related to PCOS [6–8]; if unstable, it could increase intestinal permeability by affecting intestinal metabolites, such as lipopolysaccharide, invading the systemic circulation, and causing antigen-antibody reaction in the body. An abnormal intestinal environment could activate the immune system and chronic inflammation and increase the serum insulin and androgen levels in the ovary, thereby disrupting the normal follicular development [9, 10].

According to the Fu Qing Zhu Nv Ke, an ancient TCM book in the Qing Dynasty, infertile women with obesity suffering from phlegm-dampness syndrome caused by spleen deficiency (SPSD) were treated with Buzhong Yiqi prescription (BZYQ). As previously reported, BZYQ can improve diarrhea symptoms and indirectly restore host homeostasis by recovering the gut microbiota, such as *Lactobacillus*, *Bifidobacterium*, *Enterococcus*, and *Bacillus subtilis* [11, 12]. Prescriptions based on BZYQ can significantly reduce the serum luteinizing hormone (LH), testosterone (T), fasting blood glucose (FBG), and fasting insulin (FINS) levels and the homeostatic model assessment for insulin resistance (HOMA-IR) and improve the ovarian function and pregnancy rate in patients with obesity suffering from PCOS with SPSD [13, 14]. Although BZYQ has long been applied to treat such patients, the detailed underlying mechanism remains unclear.

Metabolomics is always used conveniently to study the metabolism at the molecular level. Fecal metabolites, the cometabolism products of the gut microbiota and host, could reflect not only the status of the gut microbiota but also the relationship between commensal bacteria and the host. In PCOS, the metabolomics is mainly focused on carbohydrate, lipid, amino acid, and hormone metabolism [15]. Hence, metabolic abnormality in PCOS is not limited to the ovary; it affects systemically, thereby increasing the long-term risk of multiple diseases in patients [16]. In addition, the metabolic disorder of patients with PCOS is significantly affected by their phenotypes [17]. Although this disorder exhibits extremely distressing manifestations, the combined study of gut microbiota and fecal metabolomics in patients with obese suffering from PCOS and SPSD, especially those treated with BZYQ, is limited.

Hence, this study aimed to use 16s rRNA sequencing and nontargeted metabolomics to analyze the fecal samples of patients with obesity suffering from PCOS and SPSD and reveal the impact of BZYQ on the gut microbiota and intestinal metabolites.

## 2. Materials and Methods

### 2.1. Subjects

**Figure 1:**
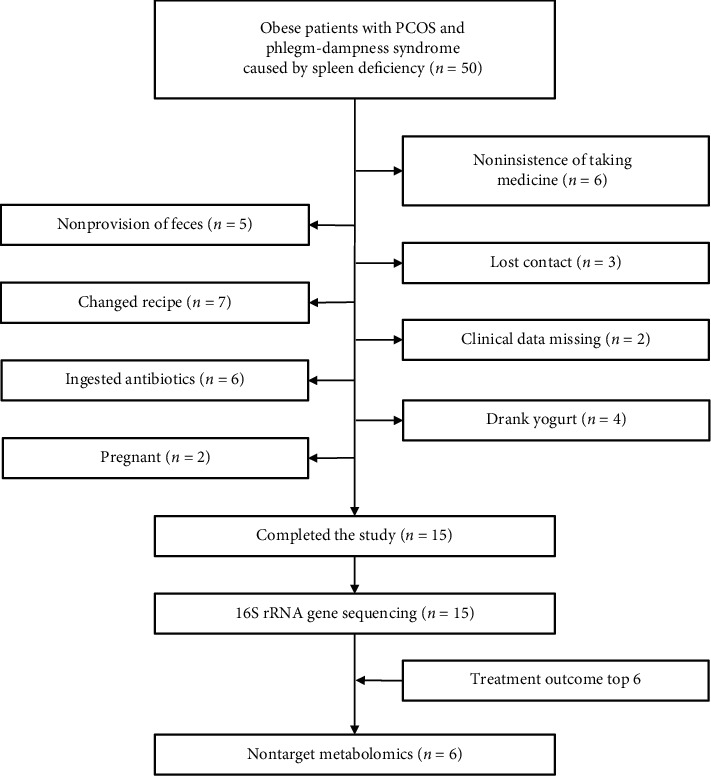
Flow chart of patients' inclusions and exclusions.

We recruited 50 patients with obesity suffering from PCOS and SPSD (BMI ≥ 28 kg/m^2^, 16–35 years old) from the TCM Gynecology Clinic of Shanghai Hospital in Shanghai between June 2016 and November 2017. However, only 15 cases completed the study by providing detailed clinical data and gut microbiota sequencing data. To explore the intestinal metabolites, we further selected six patients with the best treatment effect to completely analyze the fecal nontargeted metabolomics (Figure 1). The diagnostic criteria for obesity, PCOS, and SPSD were based on the consensus on Chinese adult obesity prevention [2], the 2003 Rotterdam Conference revisions, and the criteria set by Li et al. [18], respectively. Meanwhile, the exclusion criteria were as follows: use of oral contraceptives, antiandrogens, and insulin sensitizers in the past 3 months before the experiment; being pregnant; other known hyperandrogenemia and ovulation disorders, such as 21-hydroxylase deficiency, congenital adrenal hyperplasia, Cushing's syndrome, androgen-secreting tumors, thyroid diseases, and hyperprolactinemia; mental disorders or organic diseases; use of corticosteroids or sex steroids; history of drug and alcohol abuse in the past 2 years before the experiment; use of antibiotics, probiotics, or prebiotics in the past 3 months before the experiment; and ulcerative colitis, Crohn's disease, and other diseases that would cause dysbacteriosis. The Chinese Ethics Committee of Registering Clinical Trials approved our study (No. ChiCTRCTEC2016050), and each subject voluntarily signed the informed consent form before the trial.

### 2.2. BZYQ Preparation and Application

BZYQ mainly consisted of 30 g of Huangqi (*Hedysarum multijugum* Maxim.), 15 g of Fuling [*Poria cocos* (Schw.) Wolf], 15 g of Dangshen (*Codonopsis Radix*), 12 g of Baizhu (*Atractylodes macrocephala* Koidz.), 9 g of Shengma (*Cimicifugae Rhizoma*), 6 g of Chaihu (*Radix Bupleuri*), 9 g of Chenpi (*Citrus reticulata*), 15 g of Danggui (*Angelicae Sinensis Radix*), 15 g of Shichangpu (*Acoritataninowii Rhizoma*), 15 g of Danshen (*Radix Salviae*), 18 g of Yinyanghuo (*Epimedii Herba*), and 18 g of Shudihuang (*Rehmanniae Radix Praeparata*). The herbal medicine was obtained from Caitongdetang Pharmaceutical Co. Ltd. (Shanghai, China) and decocted at a high pressure with 2 L of cold water for 1 h to prepare the same volume and concentration of liquid medicine bags. The patients were then asked to take a liquid medicine bag at 0.5 h after meals in the morning and evening for 3 months.

### 2.3. Clinical Data and Sample Collection

On the 10th day of menstrual cycle or withdrawal bleeding, transvaginal ultrasonography was conducted to observe follicle development in the left ovary. A temperature difference of 0.3°C–0.5°C and a high temperature phase lasting for 12–14 days were considered as bidirectional body temperature, indicating ovulation. The cycle ovulation rate was calculated as follows: cycle ovulation rate = cycles of ovulation/total cycles of observation.

The samples were examined before and 3 months after BZYQ treatment. On the third day of the menstrual cycle, the levels of sex hormones such as LH, follicle-stimulating hormone (FSH), estradiol (E2), testosterone, dehydroepiandrosterone sulfate (DHEAS), and prolactin in the peripheral blood were detected in the Clinical Laboratory of Shanghai Hospital. FBG and FINS (0 h) were detected in the morning after 8 h of fasting. Next, patients ingested a pack of 75 g of glucose powder with 250 ml of warm water, and after 0.5, 1, 1.5, 2, and 3 h, the insulin levels were assessed. The area under the insulin curve (IAUC) was calculated as follows: IAUC = 1/2 × (0 h + 3 h) + 0.5 h + ¾ × 1 h + 2 h. Homeostatic model assessment for insulin resistance (HOMA-IR) was calculated as HOMA-IR = FBG × FINS/22.5 and the insulin sensitivity index (ISI) as ISI = 1/FBG × FINS. HOMA-IR ≥ 1.66 indicated insulin resistance, and ISI < 0.021 implied insulin sensitivity decrease. Fecal samples were collected 3–5 days after menstruation, but 3 days before sampling, the patients received guidance for carbohydrate-based diet (300 g/day). Approximately 10 g of fresh fecal samples was collected from each patient, using a sterile plastic spoon and a sterile test tube. Before defecation, the patients needed to clear their urine. After spreading the disposable sterile nursing pad, each patient defecated and used a sterile plastic spoon to collect the central part of the feces and place in a sterile tube. The sterile tube was then refrigerated while waiting for a laboratory personnel to take the sample. The samples were transported to the laboratory in an ice box within 2 h from sampling and stored at −80°C. Subsequently, the structure of the gut microbiota was analyzed by 16S rRNA gene sequencing, and the fecal metabolites were examined using nontargeted metabolomics.

### 2.4. Gut Microbiota Detection

Fecal samples from 15 patients were collected before and after the BZYQ treatment. Sample DNA extraction, PCR amplification, Illumina MiSeq sequencing, and post-processing of data were performed as described in previous studies [9]. The intestinal microbial diversity and changes in the abundance were analyzed using the OTU abundance data before and after BZYQ treatment, and the community composition of each sample at different classification levels was obtained. The alpha diversity was also assessed using Mothur software (v.1.30.1, https://mothur.org/) before and after BZYQ treatment. Using the R language, we drew a bar map of the community structure at the phylum and genus levels. Meanwhile, beta diversity was analyzed via principal coordinate analysis (PCoA). The differences in the species between the two groups at the phylum and genus levels were analyzed by Wilcoxon rank-sum test. Furthermore, the influence of species abundance on the differences in the gut microbiota after BZYQ treatment was evaluated by linear discriminant analysis (LDA).

### 2.5. Nontargeted Metabolomic Analysis

The nontargeted metabolomic experimental steps were based on a validated method as previously described [19]. The data were analyzed on the free online platform of Majorbio Cloud Platform (https://http://www.majorbio.com). To distinguish the overall differences in the metabolic profiles and find the different metabolites before and after BZYQ treatment (*n* = 6), we used the principal component analysis (PCA) and orthogonal partial least squares-discriminant analysis (OPLS-DA). The metabolites with variable importance for the projection (VIP) > 1 and *p* < 0.05 were considered as differential variables. The expression mode of the metabolites in each sample is displayed in the cluster heat map, and the *p* and VIP values of the metabolites are shown in a VIP bar chart. The metabolic pathway was annotated through the KEGG database (https://www.kegg.jp/kegg/pathway.html) to obtain the pathways participated by the differential metabolites. Pathway enrichment was analyzed on Python (scipy.stats), and the most relevant biological pathway was selected using Fisher's precise test.

### 2.6. Statistical Analysis

All statistical data were analyzed using SPSS software (version 21.0). The quantitative demographic and clinical data with normal distribution were analyzed by paired *t*-test and are expressed as mean with standard deviation. We employed Wilcoxon rank-sum test to analyze the quantitative sequencing data with non-normal distribution and Benjamini and Hochberg false discovery rate to check the *p* values multiple times. A double-tailed *p* < 0.05 indicated a statistically significant difference.

## 3. Results

### 3.1. Comparison of the Clinical Data of Patients with Obesity Manifesting PCOS and SPSD before and after BZYQ Treatment

**Figure 2:**
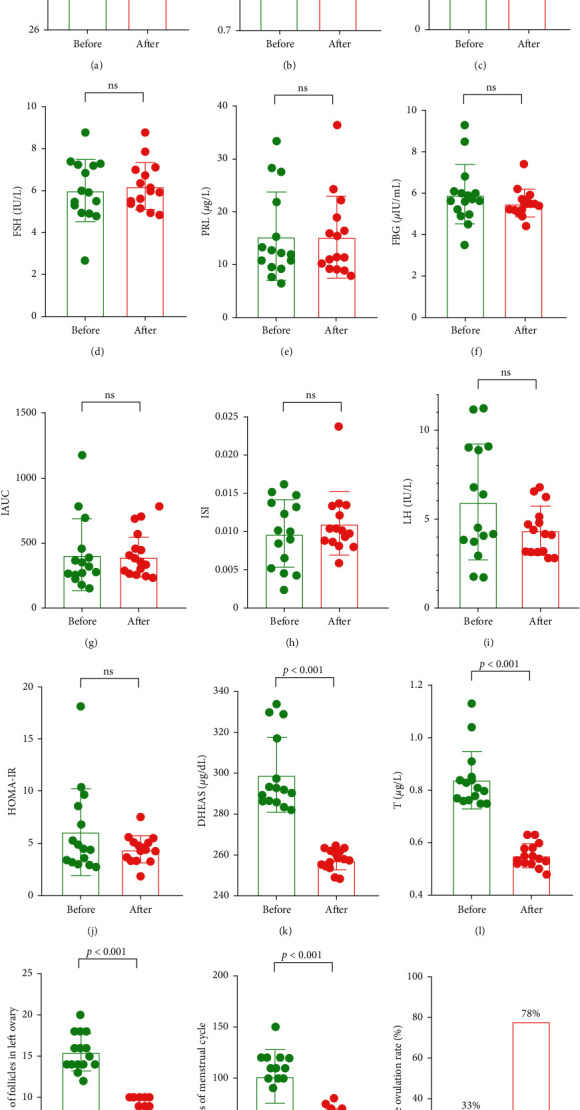
Clinical data of the patients before and after BZYQ treatment. (a) BMI, body mass index; (b) WHR, waist-hip ratio; (c) E2, estradiol; (d) FSH, follicle-stimulating hormone; (e) PRL, prolactin; (f) FBG, fasting blood glucose; (g) IAUC, insulin area under the curve; (h) ISI, insulin sensitive index; (i) LH, luteinizing hormone; (j) HOMA-IR, homeostatic model assessment for insulin resistance; (k) DHEAS, dehydroepiandrosterone sulfate; (l) T, testosterone; (m) number of follicles in the left ovary; (n) days of menstrual cycle; (o) cycle ovulation rate. ns, no significant.

Fifteen subjects completed the clinical data collection, with an average age of 27.6 (24–32) years. After BZYQ treatment for 3 months, no significant differences were found in the BMI (Figure 2(a)), waist-hip ratio (WHR) (Figure 2(b)), E2 (Figure 2(c)), FSH (Figure 2(d)), PRL (Figure 2(e)), FBG (Figure 2(f)), IAUC (Figure 2(g)), ISI (Figure 2(h)), and insulin levels (Supplementary Figure S1) compared to those before treatment. The LH (Figure 2(i)) and HOMA-IR (Figure 2(j)) indices decreased, but no statistical significance was noted. Meanwhile, the DHEAS (Figure 2(k)) and testosterone (Figure 2(l)) levels significantly decreased. In addition, BZYQ significantly reduced the number of follicles (Figure 2(m)) and improved the menstrual cycle (Figure 2(n)) and the cycle ovulation rate (Figure 2(o)).

### 3.2. Changes in the Gut Microbiota Structure

**Figure 3:**
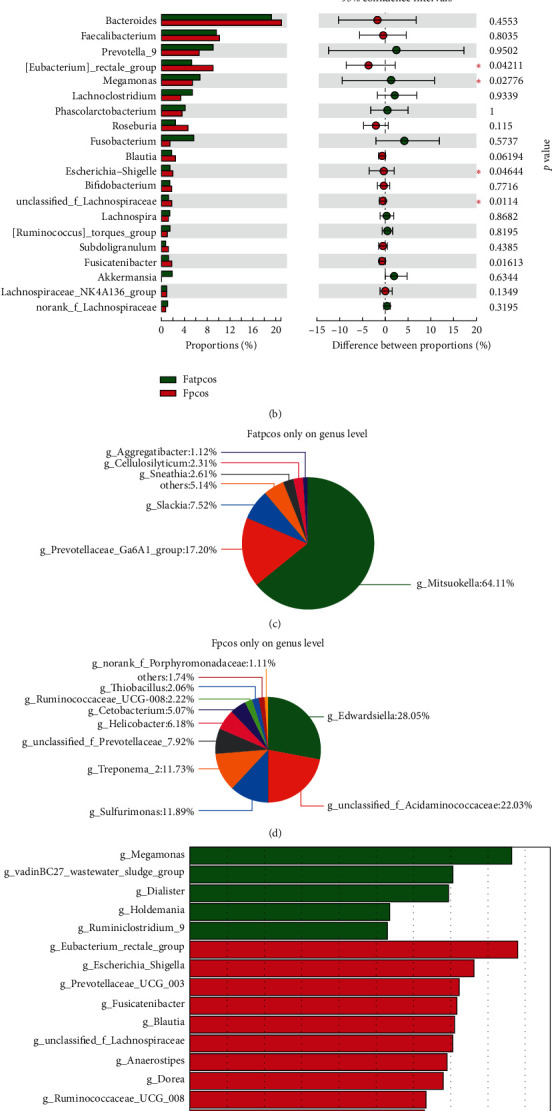
Structure of gut microbiota in patients before and after BZYQ treatment. Histogram of gut microbiota at phylum level (a) and genus level (top 20 in abundance) (b) before and after BZYQ treatment. On the left side is the mean relative abundance of species in the two groups; on the right side is the difference of species abundance before and after BZYQ treatment; boxes of different colors represent different groups. ^*∗*^, *p* < 0.05; ^*∗∗∗*^, *p* < 0.01; Fatpcos, before BZYQ treatment; Fpcos, after BZYQ treatment. Composition of abundance percentage of unique genera before (c) and after (d) BZYQ treatment. (e) Linear discriminant analysis (LDA) discriminant column chart. The greater the LDA score, the greater the impact of representative species richness on the differences between groups.

As mentioned, the structures of the gut microbiota were detected by 16S rRNA high-throughput sequencing before and after BZYQ treatment (*n* = 15). The alpha diversity (Sobs, Chao, Shannon, and Simpson) and beta diversity (PCA and PCoA) were not significantly different before and after BZYQ treatment. We detected 13 phyla, 194 genera, and 367 species before treatment and 12 phyla, 181 genera, and 344 species after treatment. At the phylum level, the BZYQ treatment increased the abundance of Firmicutes, Bacteroidetes, Proteobacteria, Actinobacteria, and Firmicutes/Bacteroidetes ratio (2.0 vs. 1.91) but decreased the abundance of Fusobacteria and Verrucomicrobia; however, no significant differences were found. Although the abundance of Spirochaetae significantly increased after BZYQ treatment (*p*=0.0006), the sample was limited and not clinically significant (Figure 3(a)).

Considering the large number of species at the genus level, we selected the top 20 species in terms of abundance for comparison. The BZYQ treatment increased the abundance of *[Eubacterium]_rectale_group*, *Escherichia-Shigella*, *unclassified_f__Lachnospiraceae*, and *Fusicatenibacter*(*p* < 0.05) but decreased the abundance of *Megamonas* (*p* < 0.05, Figure 3(b)). Before the BZYQ treatment, 26 unique genera were found, with *Mitsuokella* as the most abundant (64.11%) (Figure 3(c)). After the BZYQ treatment, 13 unique genera were found, with *Edwardsiella* as the most abundant (28.05%) (Figure 3(d)). Furthermore, the LDA scores showed that *Dialister*, *Holdemania*, *Megamonas*, *Ruminiclostridium_9*, and *vadinBC27_wastewater_sludge_group* were the characteristic genera before the BZYQ treatment and 19 genera, such as *Fusicatenibacter*, *Blautia*, and *Dorea*, were the characteristic genera after the BZYQ treatment (Figure 3(e)).

### 3.3. Comparison of Fecal Metabolites before and after the BZYQ Treatment

**Figure 4:**
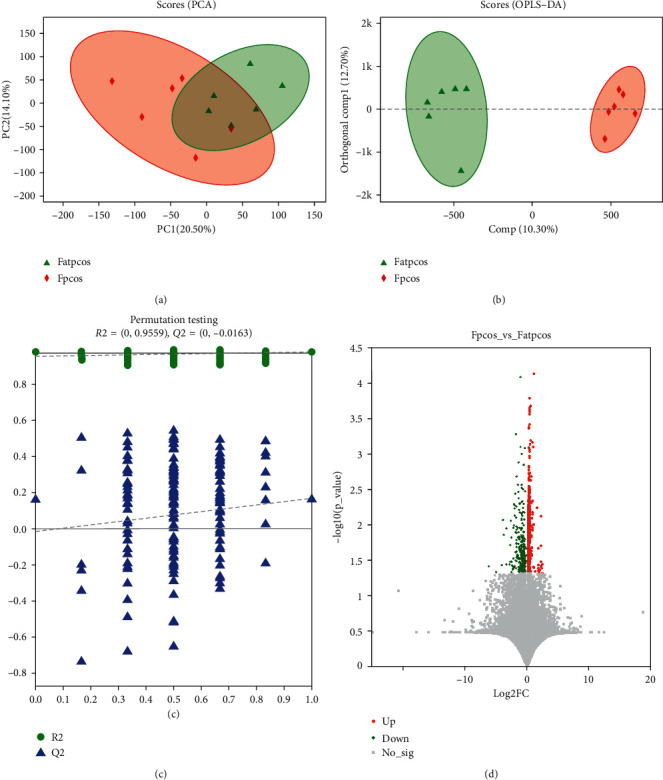
Fecal metabolites in patients before and after BZYQ treatment. PCA score chart (a) and OPLS-DA score chart (b) of fecal metabolites in obese PCOS patients before and after BZYQ treatment. Different color ellipses represent different groups. The distance between two points indicates the difference between two samples. (c) Model validation chart based on OPLS-DA of fecal metabolites in obese PCOS patients before and after BZYQ treatment. (d) Volcano map of fecal metabolites in obese PCOS patients before and after BZYQ treatment. Red indicates an increase in the level of metabolites, while green indicates a decrease in the level of metabolites. Fatpcos, before BZYQ treatment; Fpcos, after BZYQ treatment.

**Figure 5:**
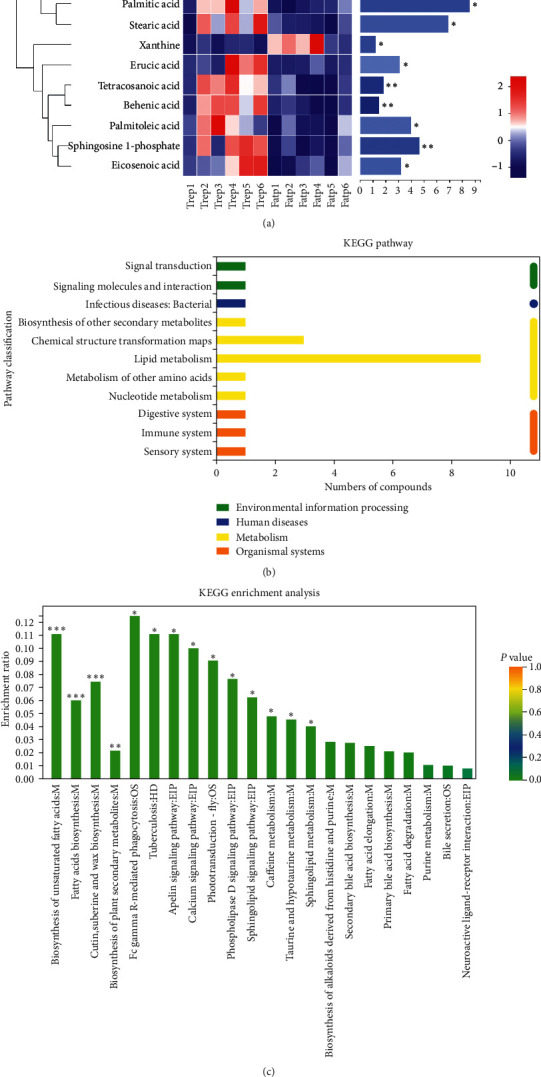
KEGG pathways related to differential fecal metabolites. (a) Heatmap of 10 differential metabolites involved in KEGG pathways. Each column represents a sample, and the bottom is the sample name; each row represents a metabolite, and the color represents the relative expression of the metabolite. On the right is the VIP bar graph of metabolites. The length of the bar represents the contribution value of the metabolites to the difference between the two groups. Trep 1–6, the samples after BZYQ treatment; Fatp 1–6, the samples before BZYQ treatment; ^*∗*^, *p* < 0.05; ^*∗∗*^, *p* < 0.01; ^*∗∗∗*^, *p* < 0.001. (b) Classification bar of KEGG pathway related to the differential metabolites. The ordinate is the name of KEGG pathway level 2, and the abscissa is the number of differential metabolites annotated to the pathway. Different colors represent different KEGG pathway level 1. (c) Enrichment of KEGG pathways related to differential metabolites. The abscissa is the name of KEGG pathway level 3; the ordinate is the enrichment rate, indicating the ratio of the number of differential metabolites in this study enriched in the pathway to the number of metabolites annotated in the pathway. The larger the ratio, the greater the degree of enrichment. ^*∗*^, *p* < 0.05; ^*∗∗*^, *p* < 0.01; ^*∗∗∗*^, *p* < 0.001.

PCA (Figure 4(a)), OPLS-DA (Figure 4(b)), and model validation (Figure 4(c)) results showed that the samples before and after BZYQ treatment (*n* = 6) were significantly grouped. We found 962 different ion peaks and 106 different metabolites (VIP > 1, *p* < 0.05) (Figure 4(d)). KEGG pathway analysis demonstrated the involvement of ten different metabolites, such as taurocholic, palmitic, and stearic acid (Figure 5(a), Table 1). The 10 differential metabolites participated in 26 KEGG pathways, with the lipid metabolism pathway containing the most differential metabolites (Figure 5(b)). The differential metabolites were significantly enriched in 14 KEGG pathways, such as the biosynthesis of unsaturated fatty acids (*p* < 0.001); fatty acids (*p* < 0.001); cutin, suberin, and wax (*p* < 0.001); and plant secondary metabolites (*p* < 0.001, Figure 5(c)).

### 3.4. Correlation Analysis among the Gut Microbiota, Fecal Metabolites, and Serum Sex Hormones after the BZYQ Treatment

**Figure 6:**
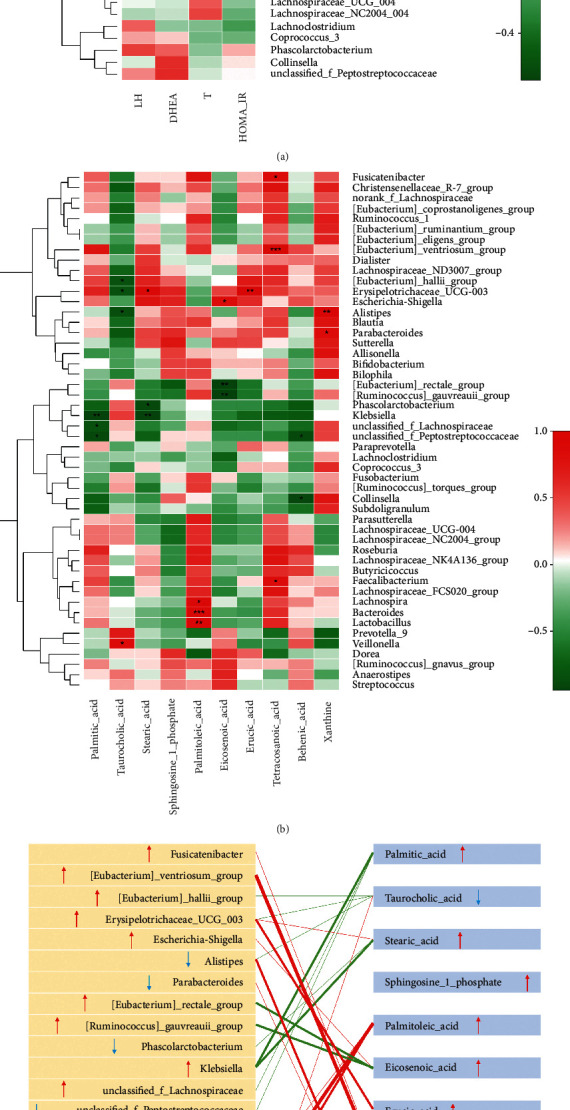
Correlation analysis among gut microbiota, fecal metabolites, and serum sex hormones. (a) Correlation heatmap of serum sex hormone, HOMA-IR, and gut microbiota (top 50) after BZYQ treatment. (b) Correlation heatmap of fecal metabolites and gut microbiota (top 50) after BZYQ treatment. Red represents positive correlation, and green represents negative correlation. (c) Correlation map of 10 differential metabolites involved in KEGG pathways and relative species at the genus level. Red line represents positive correlation, and green line represents negative correlation; the thickness of the line represents the correlation degree, and the thicker the line is, the stronger the correlation is; the arrow up indicates the increase of the abundance, and down indicates the decrease of the abundance. (d) Correlation heatmap of serum sex hormone, HOMA-IR, and 10 differential metabolites involved in KEGG pathways. ^*∗*^, *p* < 0.05; ^*∗∗*^, *p* < 0.01; ^*∗∗∗*^, *p* < 0.001.

The abundance of *Paraprevotella* had a significantly positive correlation with the serum LH level after BZYQ treatment (*p* < 0.01). The serum DHEAS level negatively correlated with *Prevotella_9* abundance (*p* < 0.05). The serum testosterone level negatively correlated with *[Eubacterium]_ruminantium_group* abundance (*p* < 0.05) but positively correlated with *Bacteroides* abundance (*p* < 0.05). In addition, HOMA-IR positively correlated with *Blautia* abundance (*p* < 0.01, Figure 6(a)).

The correlation between the 10 fecal metabolites involved in the KEGG pathways and the top 50 genera was analyzed according to their abundance. These fecal metabolites, except for sphingosine-1-phosphate, had significant correlations with specific genera (Figure 6(b)). In particular, *Bacteroides* abundance significantly correlated with the palmitoleic acid level (*p* < 0.001), and *[Eubacterium]_ventriosum_group* abundance positively correlated with the tetracosanoic acid level (*p* < 0.001, Figure 6(b)). Combined with changes in the fecal metabolites and bacterial abundance after BZYQ treatment, BZYQ had an effect on gut microbiota abundance and fecal metabolite level, showing a significant correlation (Figure 6(c)).  Table 2 summarizes the relative abundance of the abovementioned genera.

Furthermore, the tetracosanoic and palmitoleic acid level negatively correlated with the serum DHEAS level (*p* < 0.05) and serum testosterone level (*p* < 0.05), respectively, while the eicosenoic, palmitic, and erucic acid levels all positively correlated with HOMA-IR (*p* < 0.05, Figure 6(d)).

## 4. Discussion

PCOS is an endocrine metabolic disorder with multiple causes and polymorphic clinical symptoms. Clearly, TCM can improve the clinical symptoms of patients with obesity suffering from PCOS, with slight side effects, without drug dependence, and with other advantages [20]. BZYQ can effectively treat patients with obesity exhibiting PCOS and SPSD, but its mechanism on the intestinal environment remains unclear. In this study, the impact of BZYQ on the gut microbiota and fecal metabolites of such patients was discussed through the study of intestinal microecology and nontargeted metabolomics.

Gut microbiota is a general term for sojourn microorganisms in the human intestine; it has physiological functions, such as participating in the body's nutritional metabolism, antagonizing pathogenic microorganisms, promoting immunity, and maintaining the balance of the internal environment [21]. In TCM theory, the gut microbiota is closely related to the physiological function of the “spleen” [22]. When the gut microbiota is disturbed, gastrointestinal discomfort, decline in digestive and absorption function, and other clinical manifestations will occur, similar to the spleen deficiency syndrome treated with TCM [23]. Patients with obesity and PCOS often have spleen deficiency symptoms, such as fatigue, loss of appetite, and thin stool. In recent years, an increasing number of studies have confirmed that spleen-invigorating TCM compounds help regulate the gut microbiota and maintain intestinal microecology balance [24]. BZYQ is one of the spleen-invigorating TCM prescriptions with a remarkable impact on recovering the gut microbiota of the host. Gut microbiota regulation may be one of the mechanisms involved in the treatment of spleen deficiency syndrome.

In 16S rRNA high-throughput sequencing, the composition structure of the gut microbiota in patients with obesity suffering from PCOS and SPSD at the phylum and genus levels had significantly changed. At the phylum level, Spirochaetae abundance significantly increased after BZYQ treatment, but in the sample, it was too small, with slight clinical significance. At the genus level, the top 20 bacteria in terms of abundance were compared. After BZYQ treatment, the abundances of *[Eubacterium]_rectale_group*, *Escherichia-Shigella*, *unclassified_f__Lachnospiraceae*, and *Fusicatenibacter* significantly increased, whereas the abundance of *Megamonas* significantly decreased. *[Eubacterium]_rectale_group* produces butyrate (an anti-inflammatory compound) and is important in fighting inflammation [25]. Cattaneo et al. [26] found that the serum levels of proinflammatory cytokines IL-1*β*, NLRP3, and CXCL2 in the elderly with cerebral amyloidosis negatively correlated with *[Eubacterium]_rectale_group* abundance. The increased abundance of *[Eubacterium] _rectale_group* in patients with inflammatory bowel disease indicates anti-TNF-*α* enhancement [27].

PCOS is a chronic inflammatory disease, and chronic nonspecific inflammatory factors affect follicle development, influencing ovarian function, androgen synthesis in vivo, and insulin resistance; consequently, infertility and adverse pregnancy outcomes will occur [28, 29]. This study suggested that BZYQ may improve chronic inflammation in patients with obesity suffering from PCOS and SPSD by improving the abundance of *[Eubacterium]_rectale_group]*. *Megamonas* could produce short-chain fatty acids (SCFA) [30], which are converted from indigestible carbohydrates by the gut microbiota [31]. Meanwhile, den Besten et al. [32] found that SCFA could activate peroxisome proliferator-activated receptor-*γ* in the liver and muscle, thereby regulating glucose uptake and fatty acid oxidation. In addition, the gut microbiota could influence the insulin sensitivity by SCFA-mediating inflammatory responses [33]. In this study, *Megamonas* abundance decreased after BZYQ treatment. This decrement is beneficial for reducing intestinal permeability and maintaining intestinal homeostasis. In the Lefse multilevel differential analysis of species, the characteristic genera were *Dialister*, *Holdemania*, *Megamonas*, *Ruminiclostridium_9*, and *vadinBC27_wastewater_sludge_group* 5 species before the treatment, but the characteristic genera after treatment were *Fusicatenibacter*, *Blautia*, and *Dorea*. Lipopolysaccharides produced by Gram-negative bacteria are involved in the early inflammation and metabolic disease development, and these bacteria have an endotoxin effect. Gram-negative bacteria could stimulate the production of many inflammatory factors and bind to the CD14-toll receptor 4 complex on the surface of innate immune cells, resulting in chronic systemic inflammation [34]. These bacteria could also promote insulin resistance via the phosphorylation of insulin receptor substrate 1 through signaling pathways, such as nuclear factor *κ*B. Thus, *Dialister* might be associated with chronic inflammation and insulin resistance in patients with obesity manifesting PCOS and SPSD.

BZYQ also exhibited implications on the fecal metabolites of patients with such conditions. In the nontargeted metabolomic studies, taurocholic acid and xanthine were upregulated after BZYQ treatment, while eight differential metabolites, such as palmitic acid, stearic acid, and sphingosine, were downregulated. Taurocholic acid is a primary bile acid that binds amide to the amino group of the cholic acid carboxyl group and taurine. Taurocholic acid has been proven to have a significant inhibitory effect on acute and chronic inflammations, and its mechanism of action is related to its inhibition of macrophage infiltration and production of proinflammatory adipokines [35]. Palmitic acid is a long-chain saturated fatty acid, which is an important component of blood lipids. Free palmitic acid can induce stress of the endoplasmic reticulum, followed by *β*-cell apoptosis, and also inhibit insulin synthesis and secretion when glucose concentration is excessively high [36]. In the macrophages, palmitic acid upregulates the protein expression of FABP4/aP2, thereby inducing the inflammatory responses [37]. Through BZYQ treatment, palmitic acid abundance will be reduced, leading to the improvement of the disorder of glucose metabolism and chronic inflammation of patients with obesity manifesting PCOS and SPSD. Sphingosine and its metabolic enzymes are key mediators in the human body. Sphingosine kinase and its lipid product, named sphingosine-1-phosphate, are involved in signal transduction and diseases, especially in chronic inflammatory diseases and autoimmunity. These molecules are essential in the occurrence and development of the disease. In this study, the abundance of sphingosine in patients with obesity suffering from PCOS and SPSD was reduced after BZYQ treatment; hence, the inflammatory response may be reduced.

In addition, the differential metabolites were significantly enriched in 14 KEGG pathways, such as the biosynthesis of unsaturated fatty acids, fatty acids, cutin, and wax. Most of the enrichment pathways were lipid metabolism enrichment pathways. The pathogenesis of abnormal lipid metabolism is related to ApoA1 and the related regulators of lipid metabolism, adiponectin, leptin, and endogenin [38]. Obirikorang et al. [39] found that a decrease in total blood adiponectin levels can induce insulin resistance, obesity, and type 2 diabetes. Moreover, sex hormones affect the gut microbiota, which also has an impact on the serum sex hormone levels [40]. Through the analysis of the correlation of gut microbiota with fecal metabolites, serum sex hormones, and HOMA-IR values, the abundances of the different bacterial groups after BZYQ treatment were adjusted in different directions, and most of the metabolites involved in KEGG increased. Among these bacteria, *Paraprevotella* abundance in patients with obesity exhibiting PCOS and SPSD had a significantly positive correlation with the serum LH levels. A positive correlation also existed between *Blautia* abundance and the HOMA-IR values. *Blautia* contributes to maintaining glucose stability, and its dysregulation impairs the intracellular insulin signaling [41]. Furthermore, *[Eubacterium]_ventriosum_group* abundance positively correlated with tetracosanoic acid; however, this acid negatively correlated with the serum DHEAS levels. Meanwhile, *Bacteroides* abundance positively correlated with the palmitic acid and serum testosterone levels. The gut microbiota, fecal metabolite, and hyperandrogenemia demonstrated a linear relationship. Thus, BZYQ could improve the disorder of hyperandrogen by regulating the abundance of *Bacteroides*, *[Eubacterium]_ventriosum_group*, and their differential fecal metabolites.

However, this study has some limitations. First, we had not found a suitable parallel control group, thereby reducing the clinical significance of this study. Second, the treatment duration is short. PCOS is a chronic disease, and long-term management is recommended [42]. However, our study merely lasted for three sessions, and only 30% of the patients completed the trial; thus, the benefits of BZYQ may be underestimated. Third, the prescription is fixed. PCOS treatment should be adjusted according to the patient's age and ovarian reserve [43]. Our research setting will weaken the clinical effect. Fourth, selection bias existed in the study [44]. Hence, additional well-designed RCTs with appropriate controls are required.

Although the number of subjects was relatively small, the authors strictly complied by controlling the inclusion and exclusion criteria and eliminating the factors that had a great potential impact on the gut microbiota. The authors also conducted diet guidance and trained sampling method for the subjects before sampling. Hence, heterogeneity was greatly reduced in the group. Therefore, the authors are confident that the results are greatly meaningful.

## 5. Conclusions

Using fecal metabolomics combined with gut microbiota, this study explored the relationship between the intestinal environment and clinical parameters in patients with obesity suffering from PCOS and SPSD who were treated with BZYQ. BZYQ could ameliorate the serum DHEAS and testosterone levels and had an impact on the gut microbiota and metabolites in such patients. Relationships existed among the gut microbiota, fecal metabolites, and hyperandrogenism. BZYQ could ameliorate some of the endocrine disorders in these patients by regulating the abundances of *Bacteroides*, *[Eubacterium] _ventriosum_group*, and *Blautia* and the level of important fecal metabolites, such as palmitoleic, tetracosanoic, and eicosenoic acid.

## Figures and Tables

**Table 1:** Relative abundance of 10 differential metabolites involved in KEGG pathway.

Metabolite	Formula	Groups (mean with SD)		VIP	FC	*p* value	AUC (CI)
		Before (*n* = 6)	After (*n* = 6)				
Palmitic acid	C16H32O2	9490 ± 933	12219 ± 1930	8.57	0.78	0.01	0.92 (0.74–1)
Taurocholic acid	C26H45NO7S	3408 ± 2030	1106 ± 797	7.21	3.08	0.027	0.81 (0.53–1)
Stearic acid	C18H36O2	8791 ± 585	10505 ± 1413	6.90	0.84	0.021	0.86 (0.58–1)
Sphingosine 1-phosphate	C18H38NO5P	960 ± 235	1707 ± 506	4.64	0.56	0.008	0.89 (0.69–1)
Palmitoleic acid	C16H30O2	1647 ± 339	2299 ± 529	3.96	0.72	0.029	0.86 (0.64–1)
Eicosenoic acid	C20H38O2	583 ± 208	976 ± 297	3.21	0.60	0.024	0.86 (0.64–1)
Erucic acid	C22H42O2	93 ± 93	510 ± 447	3.10	0.18	0.049	0.86 (0.64–1)
Tetracosanoic acid	C24H48O2	423 ± 45	534 ± 55	1.82	0.79	0.003	0.92 (0.74–1)
Behenic acid	C22H44O2	276 ± 18	346 ± 36	1.47	0.80	0.002	0.92 (0.74–1)
Xanthine	C5H4N4O2	81 ± 49	18 ± 11	1.20	4.46	0.011	0.97 (0.9–1)

VIP, variable importance for the projection; FC, fold change (before/after); AUC, area under the curve under receiver operating characteristic curve (ROC); CI, confidence interval.

**Table 2:** Relative abundance (%) of genus in correlation analysis.

On genus level	Before (*n* = 15)		After (*n* = 15)		*p* value
	Mean	SD	Mean	SD	
*[Eubacterium]_rectale_group*	5.31	8.44	8.96	6.41	0.042
*Megamonas*	6.72	12.46	5.36	14.52	0.028
*Dialister*	0.60	1.34	0.32	0.64	0.048
*Paraprevotella*	0.22	0.40	0.63	1.51	0.47
*Prevotella_9*	8.95	22.65	6.58	16.83	0.95
*[Eubacterium]_ruminantium_group*	0.23	0.81	0.66	2.37	0.38
*[Eubacterium]_ventriosum_group*	0.20	0.29	0.58	1.03	0.14
*Blautia*	1.75	0.94	2.47	1.09	0.062
*Bacteroides*	19.15	13.26	20.99	9.82	0.455

## Data Availability

The raw clinical data of patients are not available due to hospital privacy regulations. The sequencing and LC-MS data are available on the free online platform of Majorbio Cloud Platform (http://www.majorbio.com) with the account provided by the corresponding author on reasonable request.

## Abbreviations

PCOS: Polycystic ovarian syndrome
BZYQ: Buzhong Yiqi prescription
SPSD: Syndrome of phlegm-dampness due to spleen deficiency
TCM: Traditional Chinese medicine
BMI: Body mass index
WHR: Waist-hip ratio
LH: Luteinizing hormone
FSH: Follicle stimulating hormone
E2: Estradiol (E2)
T: Testosterone
DHEAS: Dehydroepiandrosterone sulfate
P: Prolactin
FBG: Fasting blood glucose
FINS: Fasting insulin
IAUC: Area under the insulin curve
HOMA-IR: Homeostatic model assessment for insulin resistance
ISI: Insulin sensitivity index
LDA: Linear discriminant analysis
PCA: Principal component analysis
OPLS-DA: Orthogonal partial least squares-discriminant analysis
VIP: Variable importance for the projection
FDR: False discovery rate
SCFA: Short-chain fatty acids.

## Supplementary Materials

Supplementary Materials Supplementary Figure. Oral glucose tolerance test of obese PCOS patients with SPSD before and after BZYQ treatment. The insulin levels were detected 0 (A), 0.5 (B), 1 (C), 1.5(D), 2(E), and 3 (F) h after taking a pack of 75 g glucose powder with 250 mL of warm water.
