# Supplementary material for: Impact of Buzhong Yiqi Prescription on the Gut Microbiota of Patients with Obesity Manifesting Polycystic Ovarian Syndrome
Source: Evid Based Complement Alternat Med. 2021 Mar 15;2021:6671367. doi: 10.1155/2021/6671367 (PMC7984899; doi:10.1155/2021/6671367)
Supplement: Supplementary Materials — Supplementary Figure. Oral glucose tolerance test of obese PCOS patients with SPSD before and after BZYQ treatment. The insulin levels were detected 0 (A), 0.5 (B), 1 (C), 1.5(D), 2(E), and 3 (F) h after taking a pack of 75 g glucose powder with 250 mL of warm water. [file 6671367.f1.docx]

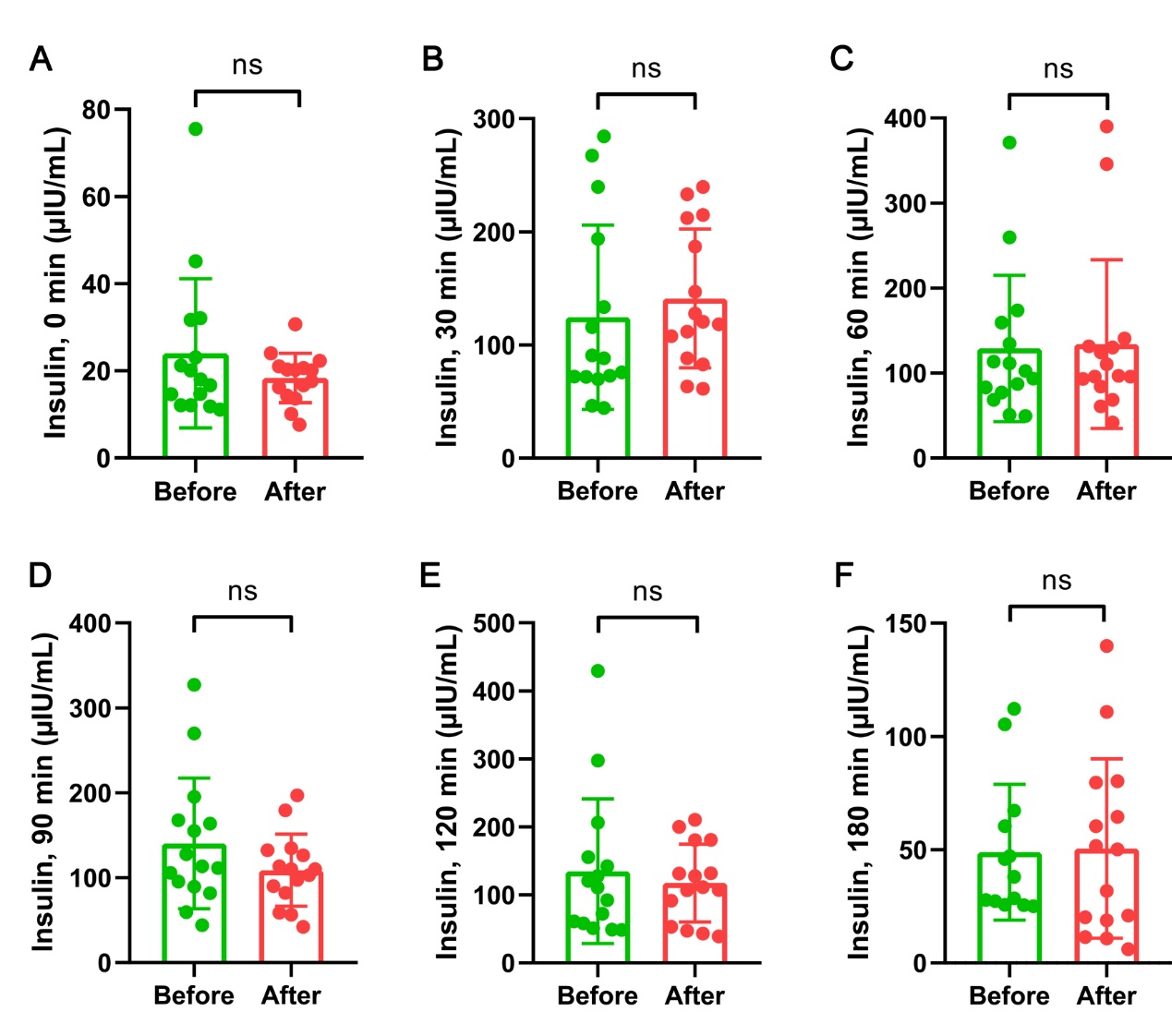


**Supplementary Figure S.** **Oral glucose tolerance test of obese PCOS patients with SPSD before and after BZYQ treatment.** The insulin levels were detected 0 (A), 0.5 (B), 1 (C), 1.5(D), 2(E), and 3 (F) h after taking a pack of 75 g glucose powder with 250 mL of warm water.
